# Association Between Parent Television-Viewing Practices and Setting Rules to Limit the Television-Viewing Time of Their 8- to 12-Year-Old Children, Minnesota, 2011–2015

**DOI:** 10.5888/pcd14.160235

**Published:** 2017-01-19

**Authors:** Martha Y. Kubik, Olga V. Gurvich, Jayne A. Fulkerson

**Affiliations:** 1 Temple University, College of Public Health, Department of Nursing, Philadelphia, Pennsylvania; 2 University of Minnesota, School of Nursing, Minneapolis, Minnesota.

## Abstract

**Introduction:**

Television (TV) viewing is popular among adults and children, and child TV-viewing time is positively associated with parent TV-viewing time. Efforts to limit the TV-viewing time of children typically target parent rule-setting. However, little is known about the association between parent TV-viewing practices and rule-setting.

**Methods:**

We used baseline height and weight data and survey data collected from 2011 through 2015 on parents and their 8- to 12-year-old children (N = 212 parent/child dyads) who were participants in 2 community-based obesity prevention intervention trials conducted in metropolitan Minnesota. Multivariable binary logistic regression analysis was used to assess the association between parent TV-viewing time on weekdays or weekend days (dichotomized as ≤2 hrs/d vs ≥2.5 hrs/d) and parent rules limiting child TV-viewing time.

**Results:**

Child mean age was 10 (standard deviation [SD], 1.4) years, mean body mass index (BMI) percentile was 81 (SD, 16.7), approximately half of the sample were boys, and 42% of the sample was nonwhite. Parent mean age was 41 (SD, 7.5) years, and mean BMI was 29 (SD, 7.5); most of the sample was female, and 36% of the sample was nonwhite. Parents who limited their TV-viewing time on weekend days to 2 hours or fewer per day were almost 3 times more likely to report setting rules limiting child TV-viewing time than were parents who watched 2.5 hours or more per day (*P* = .01). A similar association was not seen for parent weekday TV-viewing time.

**Conclusion:**

For most adults and children, a meaningful decrease in sedentariness will require reductions in TV-viewing time. Family-based interventions to reduce TV-viewing time that target the TV-viewing practices of both children and parents are needed.

## Introduction

Despite the availability and accessibility of a range of media viewing devices, television (TV) viewing remains a popular pastime among adults and school-aged youth, 8 to 12 years old. In 2013, adults spent more than half their leisure time watching TV, an estimated 2.8 hours per day (1). The 2015 Common Sense Census, a nationally representative survey of 8- to 18-year-olds that assessed media use, found that 62% of children aged 8 to 12 years reported daily TV viewing, spending on average 2.21 hours per day watching TV; black (2.59 hrs) and Hispanic (2.35 hrs) youth reported more TV-viewing time than white (2.02 hrs) youth, and youth from lower-income homes (2.30 hrs) watched more hours per day than youth from higher-income homes (1.50 hrs) (2). The amount of time spent by children watching TV is positively associated with parent TV-viewing time (3).

TV-viewing for more than 2 hours per day is associated with adverse health outcomes. For adults, this includes an increased risk of type 2 diabetes, cardiovascular disease, overweight and obesity, and all-cause mortality (4), and for school-aged youth, excess weight gain, poor fitness levels, adverse psychosocial outcomes, and decreased academic achievement (5). TV-viewing habits during childhood are also associated with obesity and poor fitness in adulthood, suggesting that interventions that aim to reduce sedentariness should start early in life (6,7).

Efforts to limit TV-viewing time by school-aged youth have typically targeted the home media environment and parent rule-setting. A 2010 Kaiser Family Foundation study found that parent rule-setting was significantly associated with less media use; however only 28% of the 8- to 18-year-old survey sample reported having rules that limited the time they could spend watching TV (8). Given the popularity of TV-viewing by adults and children alike, it is unsurprising that most children do not report having rules that limit TV-viewing time. Research on rule-based parenting practices and child TV-viewing time have often focused on parenting style (authoritarian, authoritative, or permissive) or collaborative rule-setting (9). Less is known about the association between time spent by parents viewing TV and parent rule-setting. Parents of school-aged youth are keenly positioned to influence their children’s sedentary screen time. However, success with rule-setting will likely require parents to consider their own TV-viewing practices, particularly time spent watching TV. 

We examined the association between parent weekday and weekend TV-viewing practices and parents’ use of rules to limit children’s TV-viewing time. A secondary aim was to describe characteristics of parents who reported rules limiting their children’s TV-viewing time. Results may help inform future family-focused interventions that aim to decrease child and parent sedentary screen time.

## Methods

This was a secondary data analysis of baseline data from 2 community-based randomized control trials conducted in the same large metropolitan area in Minnesota. The Healthy Home Offerings via the Mealtime Environment (HOME) Plus study evaluated the effectiveness of a family meal intervention to prevent excess weight gain among children aged 8 to 12 years with a body mass index (BMI, kg/m^2^) at or above the 50th percentile (10). The Students, Nurses, and Parents Seeking Healthy Options Together (SNAPSHOT) study, currently under way, is testing the efficacy of a healthy weight management after-school program led by school nurses to reduce excess weight gain among children aged 8 to 12 years with a BMI at or above the 75th percentile. Other inclusion criteria for both studies were English literacy and a child living with the participating parent most of the time. Exclusion criteria for HOME Plus were plans to move from the area within 6 months and medical conditions that prohibited participation (eg, extreme food allergies). Exclusion criteria for the SNAPSHOT study were only 1 child per household, moving outside the area within the next 12 months, any food allergies, physical limitations or medical conditions that would limit child’s ability to participate in physical activity, and emotional health conditions that would limit child’s ability to participate in group activities with other children. Participants for the HOME Plus study were recruited from community centers using flyers, targeted email lists, and in-person presentations and discussions; participants for the SNAPSHOT study were recruited from 2 large school districts using flyers, school and district website announcements, in-person presentations at school events, and general mailings. The total sample for this study consisted of 212 parent/child dyads (HOME Plus = 160; SNAPSHOT = 52). Data collection for the HOME Plus study occurred in the summers of 2011 and 2012; the SNAPSHOT study data collection occurred in summers of 2014 and 2015. Both studies were approved by the University of Minnesota institutional review board, and the SNAPSHOT study was also approved by the Temple University institutional review board. Across studies, participants completed written informed parent consent and child assent before baseline data were collected.

### Measures

For both studies, trained research staff collected height and weight of children and parents using standardized procedures (11). Adult BMI was calculated by dividing weight (kg) by height (m^2^); child BMI percentile was calculated using age-adjusted and sex-adjusted BMI with Centers for Disease Control and Prevention growth charts.

Parent participants in both studies completed a self-administered paper and pencil survey. Variable selection for this study was limited to the following items that were included on both surveys: parent and child demographic characteristics, parent concern about child’s weight, TV in child’s bedroom, parent rules about limiting child’s TV-viewing time and parent week day, and weekend TV-viewing time.

For demographic characteristics, we used data on child and parent age (continuous), child and parent sex (male/female), and child and parent race (dichotomized as white and nonwhite). Economic assistance was assessed with 2 questions: “Does your child receive free or reduced-priced lunches at school?” and “Does your household receive public assistance?” Responses were yes, no, and “I don’t know.” Assistance was categorized as yes if parent answered yes to one or both of the questions.

To assess parent rules limiting child’s TV-viewing time, the HOME Plus parent survey asked, “Do you have rules about how much time your child can spend watching TV or movies on any device?” (8) The SNAPSHOT parent survey asked, “Do you have rules about how much time your child spends watching TV?” (8) For both questions, response options were yes and no. For this study, a dichotomous variable (yes/no) was created. To assess parent concern about child’s weight, the HOME Plus parent survey asked, “How concerned are you about your child’s weight?” (12). Response options ranged on a 5-point Likert scale from “unconcerned” to “concerned.” The SNAPSHOT parent survey asked, “How much are you concerned about your child’s weight?” (12). Response options ranged on a 4-point Likert scale from “not concerned” to “very concerned.” For both items, responses were collapsed to create a dichotomous variable, “any concern” versus “no concern.”

To assess the presence of a TV in a child’s bedroom, the HOME Plus parent survey asked, “Does your child have a TV in his/her bedroom?” (13). The SNAPSHOT parent survey asked whether a child had any of a list of 7 types of media equipment in his or her room, of which TV was one (13). Response options for both questions were yes or no, and a dichotomous (yes/no) variable was created for this study.

To assess parent TV-viewing time, the HOME Plus parent survey asked, “On a typical weekday (Monday through Friday), how many hours do you spend watching TV or movies on any device?” (14); response options were 0, less than one half hour, 0.5 to 1 hour, 1.5 to 2 hours, 2.5 to 4 hours, 4.5 to 6 hours, or 6 or more hours. The SNAPSHOT parent survey asked, “On a typical weekday (Monday through Friday), how many hours do you spend watching TV?” (14). Response options were 0 hours, 0.5 hour, 1 hour, 2 hours, 3 hours, 4 hours, or 5 or more hours. Similar questions were asked about the weekend (Saturday or Sunday), with the same response options for each survey. For both surveys, responses were collapsed to create dichotomous variables to describe weekday and weekend parent TV-viewing time as less than or equal to 2 hours per day versus 2.5 hours or more per day.

### Statistical analysis

Descriptive statistics were calculated for variables of interest for the total sample and stratified by parent rules or no rules limiting child’s TV-viewing time. Two multivariable binary logistic regression models were fit with parent rules limiting child TV-viewing time as the dependent (outcome) variable in both models. Model 1 examined the association between parent weekday TV-viewing time (independent variable) and the outcome. Model 2 examined the association between parent weekend TV-viewing time (independent variable) and the outcome. Both models adjusted for parent race, economic assistance, child BMI percentile, and parent concern about child’s weight. Selection of the covariates was guided by prior knowledge of anticipated prognostic factors for the outcome of interest and their bivariate associations with the outcome (retained in the models at *P* < .15), data availability, and multicollinearity diagnostics. All analyses were conducted using SAS version 9.4 (SAS Institute, Inc).

## Results

Descriptive statistics for the overall sample and stratified by parent rule-setting are presented in Table 1. Among child participants, the mean age was 10 (standard deviation [SD], 1.4) years, the mean BMI percentile was 81 (SD, 16.7), approximately half were boys, and 42% were nonwhite. Most children did not have a TV in the bedroom. Among parents, the mean age was 41 (SD, 7.5) years, the mean BMI was 29 (SD, 7.5) and 36% were nonwhite. Most parents were female and did not qualify for economic assistance. Most parents were concerned about their child’s weight, and most also reported watching 2 or fewer hours per day of television on weekdays and on weekend days. 

**Table 1:** Sample Characteristics of Parent/Child Dyads, the HOME Plus Study and the SNAPSHOT Study, Minnesota, 2011–2015

Characteristic	Total Sample, N = 212^a^	Parent Rules Limiting Child Television-Viewing Time, n = 166	No Parent Rules Limiting Child Television-Viewing Time, n = 46	*P* Value^b^
**Child age, mean (SD), y**	10 (1.4)	10 (1.4)	10 (1.3)	.71
**Child BMI percentile, mean (SD)**	81 (16.7)	80 (16.8)	85 (15.9)	.07
**Parent age, mean (SD), y**	41 (7.5)	41 (7.5)	42 (7.5)	.42
**Parent BMI, mean (SD), kg/m^2^**	29 (7.5)	29 (7.9)	30 (6.0)	.65
**Child sex**				
Female	103 (49)	80 (48)	23 (50)	.83
Male	109 (51)	86 (52)	23 (50)	
**Parent sex**				
Female	198 (93)	156 (94)	42 (91)	.51
Male	14 (7)	10 (6)	4 (9)	
**Child race**				
White	123 (58)	100 (60)	23 (50)	.21
Nonwhite	89 (42)	66 (40)	23 (50)	
**Parent race**				
White	135 (64)	113 (68)	22 (48)	.01
Nonwhite	77 (36)	53 (32)	24 (52)	
**Economic assistance**				
Yes	84 (40)	70 (42)	14 (30)	.15
No	128 (60)	96 (58)	32 (70)	
**Television in child’s bedroom**				
Yes	61 (29)	45 (27)	16 (35)	.33
No	149 (71)	119 (73)	30 (65)	
**Concern for child’s weight**				
Any concern	141 (67)	120 (73)	21 (46)	.06
No concern	71 (33)	45 (27)	25 (54)	
**Parent television weekday viewing, hrs/d**				
≤2.0	159 (76)	124 (76)	35 (76)	>.99
≥2.5	50 (24)	39 (24)	11 (24)	
**Parent television weekend viewing, hrs/d**				
≤2.0	141 (67)	120 (73)	21 (46)	.001
≥2.5	70 (33)	45 (27)	25 (54)	

Abbreviations: BMI, body mass index; HOME Plus, Healthy Home Offerings via the Mealtime Environment Plus; SNAPSHOT, Students, Nurses, and Parents Seeking Healthy Options Together.

a Varies from 209 to 212 because of missing data. Baseline data from 2 community-based obesity prevention randomized control trials conducted in the same metropolitan area in Minnesota were used for analysis. Values expressed as no. (%), unless otherwise indicated.

b Fisher exact or χ^2^ tests were used to assess significance for categorical variables, and *t* tests were used to assess significance for continuous variables.

In bivariate analysis, compared with parents who did not set rules, more rule-setting parents were white (48% versus 68%; *P* = .01) and reported fewer hours of TV viewing time on weekend days (46% vs 73%; *P* = .001) (Table 1). In multivariate analysis, parents who limited their TV-viewing time on weekend days to 2 or fewer hours per day were almost 3 times more likely to report setting rules limiting child TV-viewing time than were parents who viewed 2.5 or more hours per day. A similar association was not seen among parents who limited weekday TV viewing time to 2 or fewer hours per day (Table 2). In addition, across models, parents who received economic assistance were more than twice as likely to report setting rules limiting child TV-viewing time than parents who did not receive economic assistance.

**Table 2:** Logistic Regression Analysis of the Association Between Parent Weekday and Weekend Television-Viewing Practices and Parent Rules Limiting Child Television-Viewing Time, Parent/Child Dyads, the HOME Plus Study and the SNAPSHOT Study, Minnesota, 2011–2015

Characteristic	Model 1: Parent Rules Limiting Child TV-Viewing Time, N = 209		Model 2: Parent Rules Limiting ChildTV-Viewing Time, N = 211	
	Odds Ratio (95% CI)	*P* Value	Odds Ratio (95% CI)	*P* Value
Child BMI percentile, 1-unit increase	0.99 (0.97–1.02)	.59	1.00 (0.97–1.02)	.69
**Parent race**				
Nonwhite	0.36 (0.17–0.79)	.01	0.46 (0.21–1.01)	.05
White	1 [Reference]		1 [Reference]	
**Economic assistance**				
Yes	2.60 (1.20–5.65)	.02	2.59 (1.19–5.67)	.02
No	1 [Reference]		1 [Reference]	
**Concern for child’s weight**				
Any concern	0.57 (0.24–1.40)	.22	0.59 (0.24–1.45)	.25
No concern	1 [Reference]		1 [Reference]	
**Model 1: Parent weekday television-viewing, hrs/d**				
≤2.0	0.93 (0.42–2.07)	.85	—	
≥2.5	1 [Reference]			
**Model 2: Parent weekend television-viewing, hrs/d**				
≤2.0	—		2.76 (1.35–5.64)	.01
≥2.5			1 [Reference]	

Abbreviations: —, not applicable; BMI, body mass index; CI, confidence interval; HOME Plus, Healthy Home Offerings via the Mealtime Environment Plus; SNAPSHOT, Students, Nurses, and Parents Seeking Healthy Options Together; TV, television.

## Discussion

In this study of parents with children aged 8 to 12 years, TV-viewing time by parents of 2 or fewer hours per day on weekend days but not weekdays was strongly associated with parent rule-setting to limit children’s TV-viewing time. Although most parents in this study reported 2 or fewer hours per day of TV-viewing time on weekdays (76%), fewer parents similarly limited their TV-viewing on weekend days (67%). Several studies have reported differences in TV-viewing time on weekdays versus weekend days for both children and adults that favor more TV-viewing time on weekends (15,16).

Time use studies that assess how adults and children spend their time support the idea that weekday schedules are more structured by work and longer work hours for adults and by school and school-related activities for children (1,17). By comparison, weekend days are typically less structured, for both adults and children, with more time available for leisure activities, such as sports and outdoor activities but also for TV-viewing and other sedentary activities (1,17). Parents who maintain lower levels of TV-viewing time on weekend days may be doing so despite a less structured schedule and greater choice of activities, which for most results in higher levels of TV viewing. The link between parents limiting personal TV-viewing time on weekend days and setting rules that limit child TV-viewing time may be the result of personal beliefs and knowledge about health benefits attributed to reducing screen time for self that are applied to child and family; success with personal limit-setting on weekend days that increases parental self-efficacy to similarly limit child’s TV-viewing; and effective role modeling of less TV-viewing time on weekend days that becomes the normative or usual family choice. Personal factors, such as self-efficacy and social–environmental factors that include role modeling and outcome expectancies are key constructs of Social Cognitive Theory and common targets of family-based interventions that aim to improve social support and influence health behavior (18).

The recently released and updated recommendations from the Community Preventive Services Task Force on Reducing Children’s Recreational Sedentary Screen Time found that family-based social support was the most common component of effective interventions for children aged 13 years or younger, drawing attention to the critical role of family and parent support to influence children’s sedentary screen time behavior (19,20). At the same time, the task force reported only 2 intervention studies that addressed adult screen time behavior (20). Future efforts that aim to influence child sedentary behavior will likely benefit from further development of family support strategies and an approach that recognizes the need to address parents’ screen time practices.

A secondary study finding of interest was the positive association between economic assistance and parent rule-setting. In adjusted multivariable analysis, parents who received economic assistance were more than twice as likely to report setting rules limiting child TV-viewing time than parents who did not receive economic assistance. Research indicates that youth from low-income homes report watching more hours per day of TV than do youth from high-income homes (2). It is possible that setting rules has no association with TV-viewing time among low-income youth or, conversely, that without rules TV-viewing time by low-income youth may be even higher. Further study of this association is merited with a larger and more diverse sample.

This study is among the first to assess the association between parents’ use of rules to limit children’s TV-viewing time and parent TV-viewing time, segregated by weekday versus weekend days. Other strengths were a sample that consisted of 8- to 12-year-old children and a parent (ie, dyads), with measured height and weight. Among child participants, 70% had an age- and sex-adjusted BMI at or above the 75th percentile. Studies indicate that children in the top quartile of the growth chart are at risk for excess BMI gains during the early school years, and interventions during this time to promote healthy lifestyle practices such as limiting TV-viewing time may be critical to preventing excess weight gain (21,22). Although not significant, we found that children whose parents limited TV-viewing time had on average a lower BMI percentile than children of parents who did not set rules (80th percentile vs 85th percentile, respectively; *P* = .07) . This association merits further study. Among parent participants, 65% were overweight or obese, a prevalence that mirrors the national rate (23). To date, most behavioral interventions targeting sedentary screen time have studied normal weight participants (19,20).

There are limitations to this study. The sample was a convenience sample of mostly white parents who volunteered to participate in interventions targeting healthy lifestyles. Potential heterogeneity in the estimates between the 2 samples could not be assessed because of the small sample sizes. Although very similar, the wording of survey items was not identical in the 2 studies. With the exception of height and weight, all other measures were self-reported. Because of the cross-sectional study design, causality cannot be inferred.

For most adults and children, meaningful reductions in sedentariness will require a reduction of TV-viewing time. One strategy to limit TV-viewing time for children is parent rule setting. We found that parent rule-setting to limit child TV-viewing time was significantly associated with parent TV-viewing time on weekend days but not weekdays of 2 hours or fewer per day. Family-based interventions to reduce TV-viewing time that target child and parent and the TV-viewing practices of both are needed.

## References

[1] US Department of Labor. American time use survey summary; 2014. http://www.bls.gov/news.release/atus.nr0.htm. Accessed April 30, 2016.

[2] Rideout V . The Common Sense Census: media use by tweens and teens. Common Sense Media Inc; 2015. http://www.commonsense.org/research. Accessed April 30, 2016.

[3] Jago R , Stamatakis E , Gama A , Carvalhal IM , Nogueira H , Rosado V , Parent and child screen-viewing time and home media environment. Am J Prev Med 2012;43(2):150–8. 10.1016/j.amepre.2012.04.012 22813679

[4] Grøntved A , Hu FB . Television viewing and risk of type 2 diabetes, cardiovascular disease, and all-cause mortality: a meta-analysis. JAMA 2011;305(23):2448–55. 10.1001/jama.2011.812 21673296 PMC4324728

[5] Tremblay MS , LeBlanc AG , Kho ME , Saunders TJ , Larouche R , Colley RC , Systematic review of sedentary behaviour and health indicators in school-aged children and youth. Int J Behav Nutr Phys Act 2011;8(1):98. 10.1186/1479-5868-8-98 21936895 PMC3186735

[6] Biddle SJ , Pearson N , Ross GM , Braithwaite R . Tracking of sedentary behaviours of young people: a systematic review. Prev Med 2010;51(5):345–51. 10.1016/j.ypmed.2010.07.018 20682330

[7] Mamun AA , O’Callaghan MJ , Williams G , Najman JM . Television watching from adolescence to adulthood and its association with BMI, waist circumference, waist-to-hip ratio and obesity: a longitudinal study. Public Health Nutr 2013;16(1):54–64. 10.1017/S1368980012002832 22687709 PMC10271642

[8] Rideout VJ , Foehr UG , Roberts DF . Generation M2. Media in the lives of 8 to 18 year olds. A Kaiser Family Foundation Study; 2010. http://www.kff.org. Accessed April 30, 2016.

[9] Jago R , Edwards MJ , Urbanski CR , Sebire SJ . General and specific approaches to media parenting: a systematic review of current measures, associations with screen-viewing, and measurement implications. Child Obes 2013;9(1, Suppl):S51–72. 10.1089/chi.2013.0031 23944925 PMC3746242

[10] Fulkerson JA , Friend S , Flattum C , Horning M , Draxten M , Neumark-Sztainer D , Promoting healthful family meals to prevent obesity: HOME Plus, a randomized controlled trial. Int J Behav Nutr Phys Act 2015;12(1):154. 10.1186/s12966-015-0320-3 26667110 PMC4678662

[11] Lohman T , Roche A , Martorell R . Anthropometric Standardization Reference Manual. Champaign (IL): Human Kinetics Books; 1988.

[12] Baughcum AE , Chamberlin LA , Deeks CM , Powers SW , Whitaker RC . Maternal perceptions of overweight preschool children. Pediatrics 2000;106(6):1380–6. 10.1542/peds.106.6.1380 11099592

[13] Tandon PS , Zhou C , Sallis JF , Cain KL , Frank LD , Saelens BE . Home environment relationships with children’s physical activity, sedentary time, and screen time by socioeconomic status. Int J Behav Nutr Phys Act 2012;9(1):88. 10.1186/1479-5868-9-88 22835155 PMC3413573

[14] McGuire MT , Neumark-sztainer DR , Story M . Correlates of time spent in physical activity and television viewing in a multi-racial sample of adolescents. Pediatr Exerc Sci 2002;14(1):75–86. 10.1123/pes.14.1.75

[15] Jago R , Thompson JL , Sebire SJ , Wood L , Pool L , Zahra J , Cross-sectional associations between the screen-time of parents and young children: differences by parent and child gender and day of the week. Int J Behav Nutr Phys Act 2014;11(1):54. 10.1186/1479-5868-11-54 24758143 PMC4004449

[16] Schoeppe S , Rebar AL , Short CE , Alley S , Van Lippevelde W , Vandelanotte C . How is adults’ screen time behaviour influencing their views on screen time restrictions for children? A cross-sectional study. BMC Public Health 2016;16(1):201. 10.1186/s12889-016-2789-3 26932822 PMC4774016

[17] Juster FT , Ono H , Stafford FP . Changing Times of American Youth: 1981–2003. Institute for Social Research University of Michigan; 2004. http://ns.umich.edu/Releases/2004/Nov04/teen_time_report.pdf. Accessed April 30, 2016.

[18] Bandura A . Social foundations of thought and action: a social cognitive theory. Englewood Cliffs (NJ): Prentice-Hall; 1986.

[19] Community Preventive Services Task Force. Reducing children’s recreational sedentary screen time. Recommendation of the Community Preventive Services Task Force. Am J Prev Med 2016;50(3):416–8. 10.1016/j.amepre.2015.09.014 26897343

[20] Ramsey Buchanan L , Rooks-Peck CR , Finnie RKC , Wethington HR , Jacob V , Fulton JE , ; Community Preventive Services Task Force. Reducing recreational sedentary screen time. A community guide systematic review. Am J Prev Med 2016;50(3):402–15. 10.1016/j.amepre.2015.09.030 26897342 PMC9664246

[21] Datar A , Shier V , Sturm R . Changes in body mass during elementary and middle school in a national cohort of kindergarteners. Pediatrics 2011;128(6):e1411–7. 10.1542/peds.2011-0114 22106078 PMC3387897

[22] Nader PR , O’Brien M , Houts R , Bradley R , Belsky J , Crosnoe R , ; National Institute of Child Health and Human Development Early Child Care Research Network. Identifying risk for obesity in early childhood. Pediatrics 2006;118(3):e594–601. 10.1542/peds.2005-2801 16950951

[23] Ogden CL , Carroll MD , Kit BK , Flegal KM . Prevalence of childhood and adult obesity in the United States, 2011–2012 JAMA 2014;311(8):806-14. 10.1001/jama.2014.732 PMC4770258 24570244

